# Cross-vendor reliability of functional and structural brain connectivity in a travelling cohort

**DOI:** 10.1038/s41598-026-47705-1

**Published:** 2026-04-10

**Authors:** Lionel Butry, Johanna Thomä, Johannes Forsting, Elena K. Enax-Krumova, Lara Schlaffke

**Affiliations:** 1 Ruhr University Bochum, BG University Hospital Bergmannsheil, Department of Neurology, Bochum, Germany; 2 Department of Information Technology, FH Dortmund, University of Applied Sciences and Arts, Dortmund, Germany; 3 Ruhr University Bochum, St. Josef Hospital, University Hospital of Pediatrics and Adolescent Medicine, Bochum, Germany

**Keywords:** Generalizability, Magnetic resonance imaging, Harmonisation, fMRI, DTI, Connectome, Medical research, Neurology, Neuroscience

## Abstract

Scanner-related bias is a major challenge in multi-centre neuroimaging studies. This study assesses cross-vendor reliability of functional and structural brain connectivity (FC, SC) and evaluates the effect of neuroComBat harmonisation. A travelling cohort (*n* = 10) of healthy participants was scanned on a Siemens Prisma 3T and a Philips Achieva 3.0T system within one week. Reliability metrics of FC (Pearson’s r) and SC (based on *iFOD2* & *SIFT2*) were calculated for each edge before and after neuroComBat harmonisation. At the subject-level, reliability was poor for FC (ICC: 0.22 ± 0.23, wsCV: 62 ± 14%, unharmonised) and fair for SC (ICC: 0.43 ± 0.32, wsCV: 28 ± 15%, unharmonised). At the group-level, Bland-Altman analyses showed minimal systematic bias between scanners in both modalities, but with wide limits of agreement. Pattern similarity was 0.63 (FC) and 0.98 (SC). G Theory analyses showed the scanner accounted for 11.8% (FC) and 7.9% (SC) of the variance. After harmonisation, subject-level reliability only improved marginally, but scanner-dependent variability dropped to 0.8% and 1.1% in FC and SC, respectively. Overall, reliability was higher for SC than FC mirroring their differential temporal stability, for group-averaged than subject-level data, and for full-connectome organisation than for individual edges. NeuroComBat worked as designed, successfully mitigating scanner-dependent variance at the group but not the subject level.

**Supplementary Information:**

The online version contains supplementary material available at 10.1038/s41598-026-47705-1.

## Introduction

Mapping the human brain as a complex network of interconnected regions, known as the human connectome, has become a cornerstone of systems neuroscience^1^. Using magnetic resonance imaging (MRI), a connectome can be either characterised by functional connectivity (FC), the statistical synchronicity between brain regions measured with resting-state functional MRI (rs-fMRI) or by structural connectivity (SC), which captures the physical white-matter pathways linking brain regions as measured with diffusion-weighted MRI (dMRI)^2^. Thus, functional and structural connectomes provide complementary insights into the brain’s intrinsic organisation and have enabled investigations at the level of brain networks rather than isolated regions.

Multi-centre neuroimaging studies have become increasingly common as they enable the recruitment of larger and more diverse cohorts, the replication of findings across sites, and the integration of data from independent studies into large-scale consortia^3,4^. However, an enduring challenge in multi-centre research is scanner-related variability^5,6^. Even when acquisition parameters are harmonised, systematic differences may persist between scanner models, manufacturers and hardware configurations^7^. These differences can substantially bias quantitative MRI measures by confounding biological inferences or inflating inter-subject variance, thereby obscuring meaningful group-level findings or subject-level longitudinal investigations. This problem arises not only for multi-centre studies but for single centres facing a scanner switch – an important issue and real-life scenario for studies with long acquisition timeframes. Hence, cross-manufacturer reproducibility of functional and structural connectomes is a critical prerequisite for reliable cross-scanner analyses.

In connectomics, region-to-region connectivity represents the most fundamental analytical level. Each connection (‘edge’) between two regions defines the elementary unit from which e.g. complex network patterns or graph-theoretical measures are derived. Assessing edge-level reproducibility is therefore essential, as variability at this level can propagate to higher-level analyses.

One approach to mitigate scanner effects are statistical harmonisation techniques. Instead of adjusting the images themselves, these techniques correct the features extracted from them (i.e. SC or FC). A widely used harmonisation method for extracted features is *ComBat*, which was initially developed for genomic data^8^ but has been adapted to neuroimaging (*neuroComBat*)^9^. By modelling non-biological variance while preserving the true biological signal, *neuroComBat* has shown promise in reducing scanner-related bias in various imaging modalities^7,9–11^.

The present study aims to (1) assess cross-vendor reliability of functional and structural region-to-region connectivity between a Siemens Prisma 3T and a Philips Achieva 3.0T system in healthy participants, and (2) evaluate the influence of *neuroComBat* harmonisation on the reliability. By quantifying reproducibility across multiple reliability metrics, this work provides a comprehensive analysis of scanner effects on the human functional and structural connectome.

## Methods

For the reporting of this study, the ‘Guidelines for Reporting Reliability and Agreement Studies (GRRAS)’ were used (**Suppl. Table 1**)^12^.

### Study design and participants

An MRI reliability study employing a travelling cohort was conducted at two imaging facilities within the Research Department of Neuroscience, Ruhr University Bochum, Germany, utilising a Siemens Prisma 3T and a Philips Achieva 3.0T MRI system.

A total of ten healthy young adults were recruited through word of mouth in the Ruhr area, Germany. Study size was limited by feasibility. To ensure a homogeneous sample, recruitment targeted a balanced sex ratio (1:1, male-to-female) and an age standard deviation of under five years. Participants were excluded if they fulfilled any of the following criteria: (1) psychiatric or neurological diseases, and (2) absolute MRI contraindications such as metal implants or severe claustrophobia. All participants provided written informed consent. Ethical approval was obtained from the local ethics committee of the Medical Faculty of the Ruhr University Bochum (23-7829-BR), and all methods were performed in accordance with the relevant guidelines and regulations, including the Declaration of Helsinki.

### MRI acquisition

Each participant was scanned using a 3.0T Achieva MRI system (Philips Healthcare, Best, The Netherlands) with a 32-channel head coil and a 3T Magnetom Prisma MRI system (Siemens Healthineers, Munich, Germany) with a 64-channel head coil. As anatomical reference, a high-resolution T1-weighted (T1w) scan was acquired, followed by a rs-fMRI and a multi-shell dMRI scan (Table 1). During the rs-fMRI scan, participants were instructed to close their eyes, keep awake and let their minds wander without focusing on anything specific. The scanner hardware and software configurations remained unchanged throughout the data acquisition period. The used sequences reflect a real-life scenario, that may occur, when an MRI system is replaced without the application of specific clinic or research keys or pulse programming.

**Table 1:** Summary of the acquisition protocols across modalities and scanners.

	Philips Achieva TX 3.0T	Siemens Prisma 3T (Syngo MR XA30)
T1w		
Pulse sequence	MP-3D GRE	MP-3D GRE
TR	7.7 ms	1650 ms
TE	3.6 ms	2.98 ms
Flip angle	8°	8°
Matrix size	240 × 240	256 × 256
No. of slices	220	176
Slice gap	0 mm	0 mm
Resolution	1 × 1 × 1 mm^3^	1 × 1 × 1 mm^3^
Duration	4 min 1 s	3 min 57 s
rs-fMRI		
Pulse sequence	GE-EPI	GE-EPI (cmrr_mbep2d_bold)
TR	2500 ms	2500 ms
TE	30 ms	30 ms
Flip angle	90°	90°
Matrix size	80 × 80	80 × 80
No. of slices	47	48
Slice gap	0 mm	0 mm
Resolution	3 × 3 × 3 mm^3^	3 × 3 × 3 mm^3^
No. of volumes	190	190
Acceleration	SENSE-3	MB-2; GRAPPA-4
Duration	~ 7 min 55 s	~ 7 min 55 s
dMRI		
Pulse sequence	SE-EPI	SE-EPI (cmrr_mbep2d_diff)
TR	10,000 ms	3800 ms
TE	91 ms	90 ms
Flip angle	90°	90°
Matrix size	112 × 112	110 × 110
No. of slices	61	62
Slice gap	0 mm	0 mm
Resolution	2 × 2 × 2 mm^3^	2 × 2 × 2 mm^3^
No. of volumes	117	117
Acceleration	SENSE-2.5	MB-2; GRAPPA-4
B values	0; 1000; 1800; 2500	0; 1000; 1800; 2500
No. gradient directions	12; 30; 30; 45	12; 30; 30; 45
Duration	~ 19 min 30 s	~ 7 min 25 s

dMRI = diffusion-weighted magnetic resonance imaging. T1w = T1-weighted. rs-fMRI = resting-state functional magnetic resonance imaging. TE = echo time. TR = repetition time.

### Preprocessing of MRI data

The rs-fMRI and T1w data were preprocessed in accordance with the methodology described in^13^. In short, fMRIPrep v23.2.0^14^ was utilised to apply intensity non-uniformity correction, skull-stripping and segmentation into cerebrospinal fluid (CSF), white matter, and grey matter tissue maps to the T1w images.

The rs-fMRI images were slice-time corrected and coregistered to the participant’s native T1w space acquired on the same scanner. Several confounding time series were calculated using CompCor on the preprocessed rs-fMRI data, except for head-motion parameters, which were estimated before any spatiotemporal filtering. Time series was filtered to a bandwidth of 0.008 to 0.1 Hz, nuisance signals from 24 motion parameters and 8 white matter and CSF tissue parameters were regressed out, and volumes with high motion (framewise displacement [FD] > 0.5 mm) were discarded, as recommended for resting-state FC by a benchmarking study^15^. No global signal regression was applied. Finally, the signal was z-score standardised. A predefined exclusion criterion was applied whereby datasets with more than 30% of volumes scrubbed would be discarded; however, no dataset reached this threshold.

The dMRI pipeline followed the quantitative structural connectivity framework implemented in MRtrix3 v3.0.3^16^. Initial preprocessing of dMRI data was performed as described in detail in^13^, including denoising, correction for Gibbs ringing, motion artefacts, eddy currents, inhomogeneities and bias field. The multi-shell multi-tissue constrained spherical deconvolution (MSMT-CSD) model was utilised to obtain fibre orientation distributions (FODs)^17^. The response function for FOD computation was computed for each participant individually. Multi-tissue informed intensity normalisation was performed on the FODs. The probabilistic tractography algorithm *iFOD2* (Streamlines 5,000,000; Step size 1; Angle 45°; Minimum track length 4 mm; Maximum track length 250 mm; FOD amplitude cutoff 0.05; backtracking enabled) was used within the anatomically-constrained tractography (ACT) framework^18^. The ACT approach enhances the biological plausibility of streamline reconstruction by restricting tract generation to the white matter mask and defining the grey-to-white-matter interface as both seed and terminal region. Accordingly, the preprocessed T1w images were coregistered to each participant’s native dMRI space using b-spline nonlinear transformations (*antsRegistrationSyNQuick.sh*, ANTs).

### Connectome construction

The Brainnetome atlas, which is based on both functional and structural connectional architecture, was used to parcellate the brain into 246 regions, including 210 cortical and 36 subcortical regions^19^. Hence, the functional and structural connectome consists out of 30,135 unique connections.

For FC, NiLearn’s *ConnectivityMeasure* was utilised to compute the Pearson’s correlation coefficient between the BOLD time series of each atlas region. Lastly, Fisher’s r-to-z transformation was applied to the values. Hence, FC can be interpreted as the amount of synchronised brain activity between two regions.

For SC, first the *SIFT2* filtering method (*tcksift2*, MRtrix3) was applied to the probabilistic tractogram to assign a weight to each streamline to match the tractogram to the underlying FOD magnitudes^20^. Secondly, *tck2connectome* (MRtrix3) was used to compute the sum of streamline weights which connect each atlas region. With doing so, the SC is proportional to the effective cross-sectional area of white matter axons connecting two regions^20^.

### Statistical analysis

All analyses were performed using R v4.5.1 (www.r-project.org). Functional and structural connectivity were investigated separately.

#### Harmonising data

Harmonisation was performed separately for FC and SC using the R package *neuroComBat* v1.0.14 with the ‘scanner vendor’ as batch variable. Covariates included in the harmonisaton were ‘age’ and ‘sex’, ‘days between scans’ and ‘daytime of scan’. The default settings of *neuroComBat* were used. All analyses stated below were conducted both on the unharmonised and harmonised dataset.

#### Reliability assessment

Reliability metrics were computed at the region-to-region (edge) level. For visualisation purposes, edge-level reliability metrics were aggregated based on seven major anatomical regions (namely frontal lobe [Fro], insular cortex [Ins], limbic regions [Lim], occipital lobe [Occ], parietal lobe [Par], subcortical regions [Subc], temporal lobe [Tem]) or eight large-scale functional brain networks (based on Yeo7 atlas + subcortical regions, namely central attention network [CEN], dorsal attention network [DAN], default mode network [DMN], limbic network [LIM], somatomotor network [SMN], subcortical regions [SUBC], ventral attention network [VAN], visual network [VIS]), where applicable.

To assess cross-vendor reliability at the subject-level, the intraclass correlation coefficient (ICC) and within-subject coefficient of variation (wsCV) were used (Suppl. Table 2). The ICC was computed using the *irr* R package, specifying a two-way random-effects model for consistency and single-unit. Higher ICC values indicate greater reliability, with values < 0.5 considered poor, 0.5 – 0.75 moderate, 0.75 – 0.9 good, and > 0.9 excellent^21^. In case of negative ICC estimates, they were set to zero^22^. The wsCV was calculated for each subject as the standard deviation across scanners divided by the mean of absolute connectivity values across scanners, expressed as a percentage, and then averaged across subjects. Lower wsCV values indicate lower relative variability between scanners, thus higher reliability.

To assess cross-vendor reliability at the group-level, Bland-Altman analysis was conducted. Additionally, the Pearson correlation coefficient (PCC) was calculated on the vectorised full connectome and on the network/lobe-level to assess the pattern similarity of connectivity^23^. Higher PCC indicates higher pattern similarity.

Lastly, components of measurement error were investigated in accordance with the Generalizability Theory (G-Theory) approach. G-Theory can decompose the single error component of classical test theory into multiple components^24^. A one-facet analysis design was conducted: First, variance components were estimated for the object of measurement (i.e. participant, $\:{\sigma\:}_{p}^{2}$) and the facet of the measurement (i.e. scanner, $\:{\sigma\:}_{s}^{2})$. The residual variance ($\:{\sigma\:}_{ps,e}^{2}$) combines the interaction between participant and scanner as well as random error. Variance components were estimated using a linear mixed-effects regression model (*lme4* R package) with all factors modelled as random intercept effects, resulting in the following equation:

$$\:{\sigma\:}^{2}\left({X}_{ps}\right)={\sigma\:}_{p}^{2}+{\sigma\:}_{s}^{2}+{\sigma\:}_{ps,\:\:e}^{2}$$

The variance components were reported as percentages of the overall variance.

## Results

### Participants

Ten healthy adults (mean age: 27.2 ± 3.1 years; mean BMI: 23.4 ± 3.5 kg/m²) were successfully recruited and scanned at both scanner sites. The mean duration between scans was 5 ± 2.67 days (range: 0–7). Among the participants, 40% (*n* = 4) had never undergone an MRI, 30% (*n* = 3) had a prior cranial MRI, and 30% (*n* = 3) had an MRI performed on a non-cranial body region. A detailed overview of participant characteristics is provided in Suppl. Table 3. There were no adverse events during MRI acquisition.

### Subject-level reliability

**Fig. 1:**
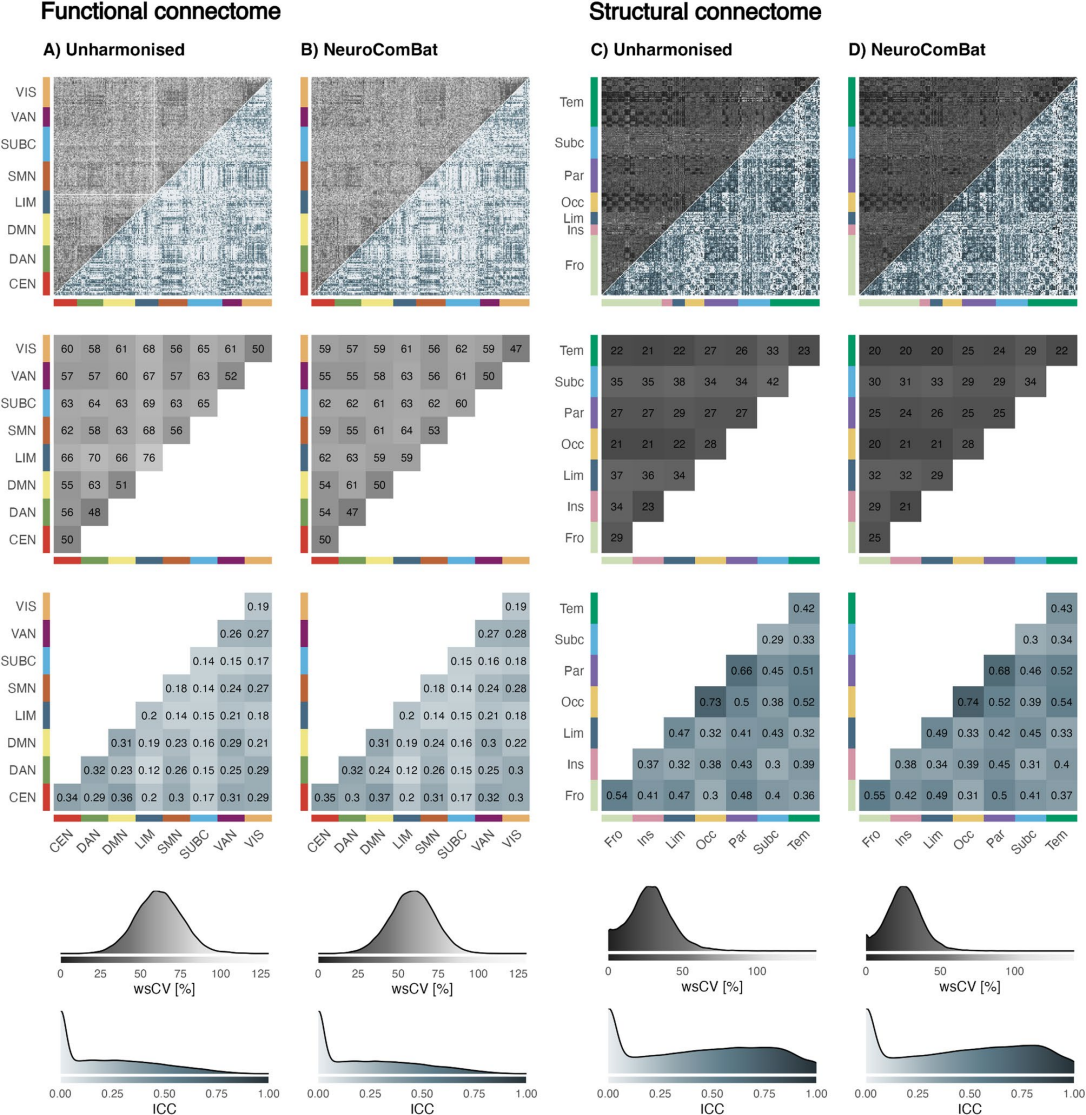
Subject-level reliability of the functional (**A**,**B**) and structural (**C, D**) connectome measured with the within-subject coefficient of variation (wsCV, black, range: 0–125%) and the intraclass correlation coefficient (ICC, blue, range: 0–1). Heatmaps: Upper triangle contains wsCV values and lower triangle contains ICC values. Darker colours represent higher reliability. First row: Heatmap axes represent brain regions of the Brainnetome atlas, ordered by large-scale brain networks or major anatomical regions. Second and third row: Edge-level heatmap aggregated by large-scale brain network or major anatomical region. Fourth and fifth row: Density plot of edge-level wsCV and ICC with integrated colour scale.

In the unharmonised functional connectome, subject-level cross-vendor reliability was poor, with mean ICC values across the full connectome of 0.219 ± 0.226 (range: 0–0.93) and mean wsCV of 62 ± 14% (range: 13–128%) (Fig. 1A). Application of *neuroComBat* to the functional connectome yielded no meaningful improvement, with subject-level reliability remaining poor (ICC: 0.223 ± 0.233, wsCV: 59 ± 14%, Fig. 1B). FC edges involving the LIM and SUBC showed the lowest reliability, whereas those within and between the CEN, DAN, and DMN were relatively higher, although they remained within the poor reliability range.

In the unharmonised structural connectome, subject-level cross-vendor reliability was fair, with mean wsCV across the full connectome of 28 ± 15% (range: 0–119%) and mean ICC values of 0.425 ± 0.322 (range: 0–1) (Fig. 1C). After *neuroComBat* harmonisation, subject-level reliability remained fair, with a slight reduction in mean wsCV (25 ± 12%) and a marginal increase in mean ICC (0.439 ± 0.328) (Fig. 1D).

### Group-level reliability

**Fig. 2:**
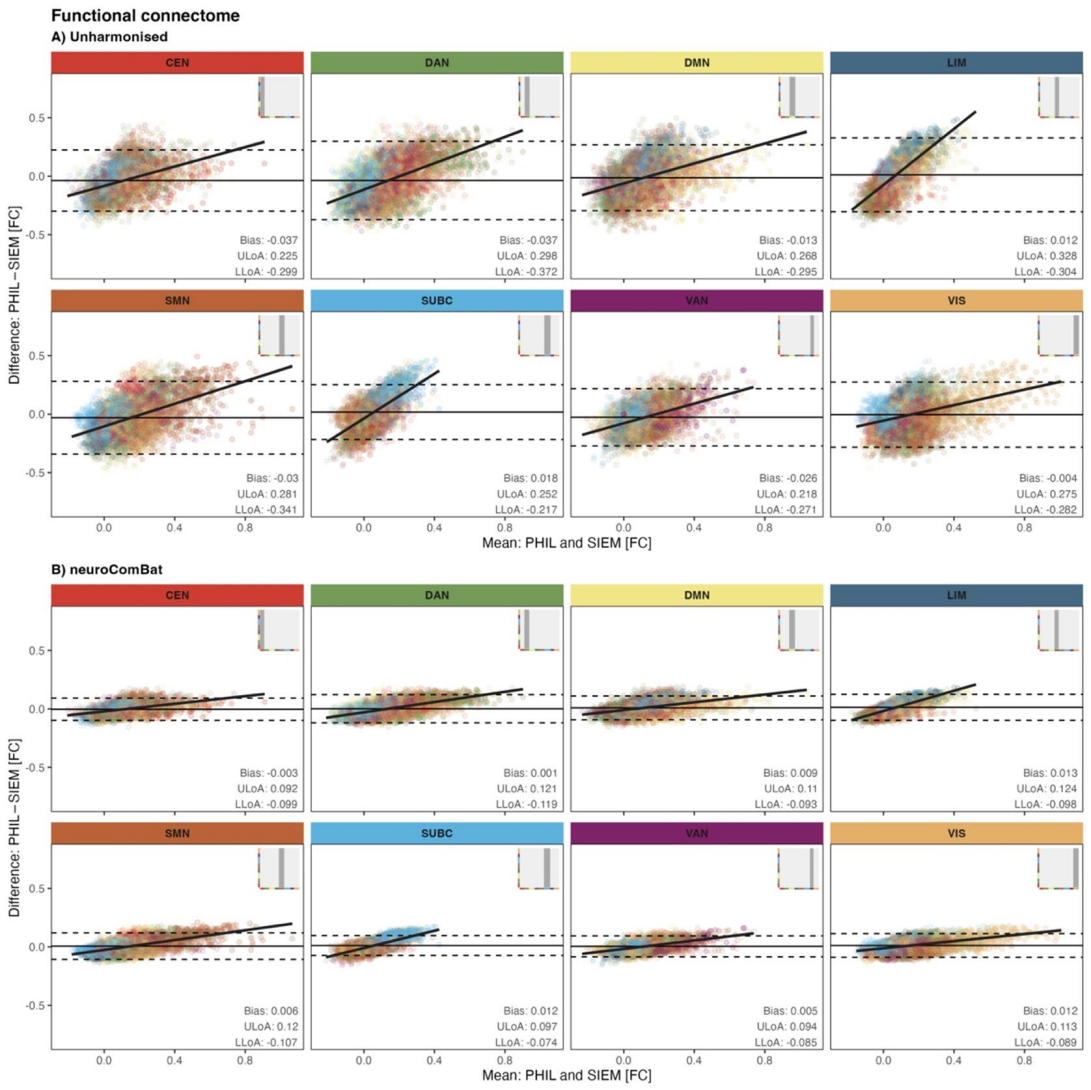
Bland-Altman plots for the functional connectome by large-scale brain network: (**A**) Results before harmonisation. (**B**) Results after neuroComBat harmonisation.

In the unharmonised functional connectome, Bland-Altman analysis, faceted by large-scale brain networks, showed mean FC differences between − 0.037 and 0.018, indicating minor systematic differences between Philips and Siemens MRI systems across the full functional connectome (Fig. 2A). However, the limits of agreement were wide (LLoA: − 0.372 to − 0.217, ULoA: 0.218 to 0.328, Fig. 2A), indicating considerable variability in scanner-related differences at the level of individual edges.

A proportional bias was evident by positive regression slopes in the Bland-Altman plots across all networks, with the steepest slopes involving SUBC and LIM edges (Fig. 2A). This suggests that scanner agreement depended on the magnitude of FC: the stronger the FC (i.e., further from zero), the larger the disagreement between the scanners, with Siemens yielding weaker FC values (closer to zero).

After *neuroComBat* harmonisation, mean FC differences were reduced (range: − 0.003 to 0.013) and LoA were smaller (LLoA: − 0.119 to − 0.074, ULoA: 0.092 to 0.124), indicating reduced variability in scanner-related differences at the level of individual edges (Fig. 2B). Additionally, regression slopes were smaller, indicating a reduction in proportional bias (Fig. 2B).

**Fig. 3:**
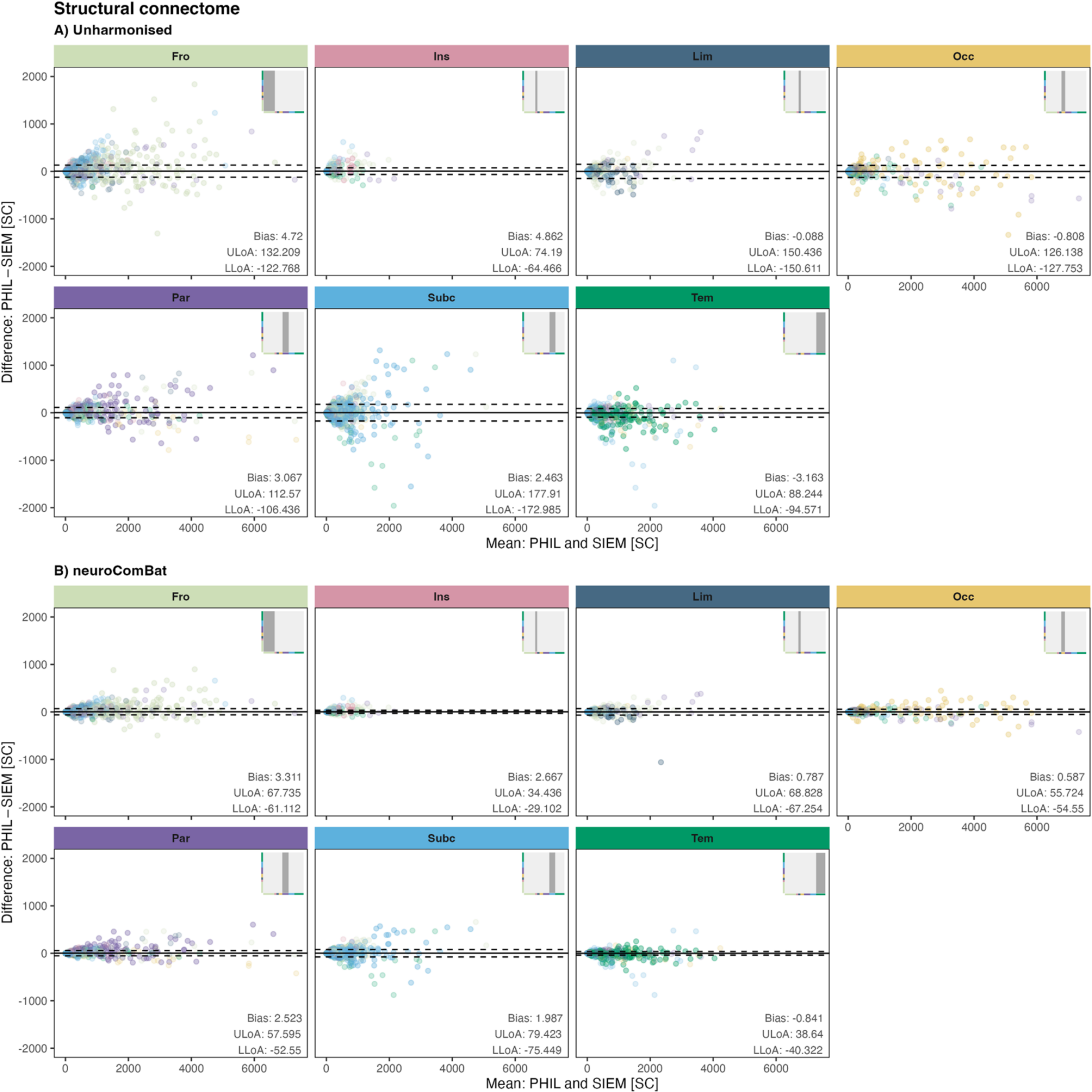
Bland-Altman plots for the structural connectome by anatomical region: (**A**) Results before harmonisation. (**B**) Results after neuroComBat harmonisation.

In the unharmonised full structural connectome, Bland-Altman analysis, faceted by brain anatomical regions, showed mean SC differences between − 3.2 and 4.9, indicating minimal systematic bias between Philips and Siemens MRI systems (Fig. 3A). However, compared to the scale of the SC values, limits of agreement were relatively wide (ULoA: 74.2 to 177.9, LLoA: − 64 to − 173, Fig. 3A), indicating moderate variability in scanner-related differences at the level of individual edges. Additionally, several edges fall outside the limits of agreement, predominantly edges within the same anatomical region.

When applying *neuroComBat* to the structural connectome, SC mean differences were reduced (range: − 0.8 to 3.3) and LoA narrowed (LLoA: − 29.1 to − 75.4, ULoA 34.4 to 79.4), indicating reduced variability in scanner-related differences at the level of individual edges (Fig. 3B).

### Connectivity pattern similarity

**Fig. 4:**
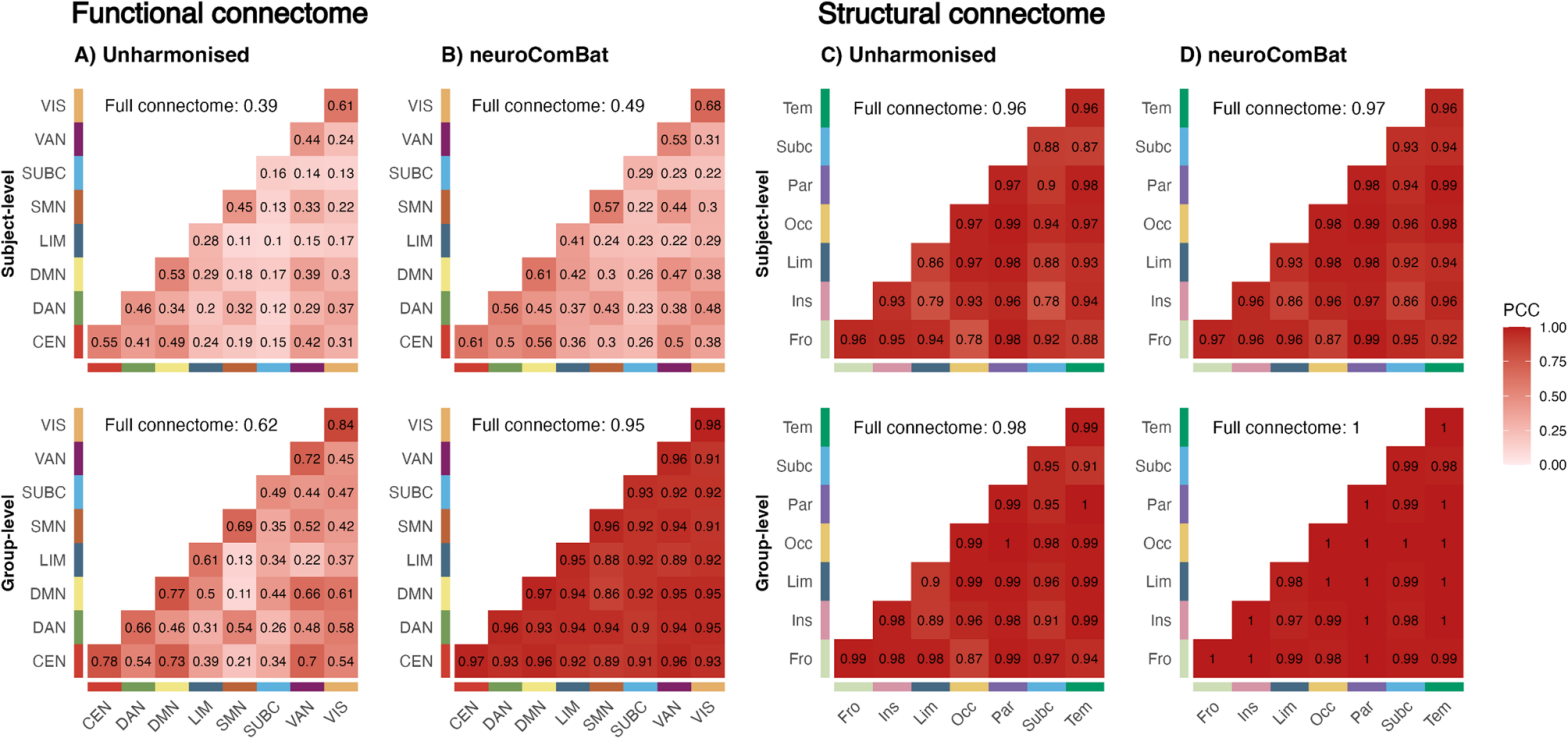
Connectivity pattern similarity of the functional (**A**,**B**) and structural (**C**,**D**) connectome measured by Pearson correlation coefficient (PCC). First row: subject-level pattern similarity. Second row: group-level pattern similarity.

In the unharmonised functional connectome, the similarity in the pattern of the full functional connectome between the two scanners was moderate at the group-level (PCC: 0.62) but low at the subject-level (PCC: 0.39), as shown in Fig. 4A. Interestingly, similarity was higher in within-network FC patterns (mean group-level PCC: 0.70 ± 0.11) compared to between-network FC patterns (mean group-level PCC: 0.43 ± 0.16) (Fig. 4A). This was also evident at the subject-level. After *neuroComBat* harmonisation, group-level FC pattern similarity increased drastically (full connectome PCC: 0.95) but only increased moderately at the subject-level (full connectome PCC: 0.49) (Fig. 4B).

In the unharmonised full structural connectome, the pattern similarity between the two scanners was excellent at the group (PCC: 0.98) and subject-level (PCC: 0.96), as provided in Fig. 4C. When applying *neuroComBat* to the structural connectome, group and subject-level SC pattern similarity further increased (full connectome PCC: 1 and 0.97, Fig. 4D).

### Sources of variability

**Fig. 5:**
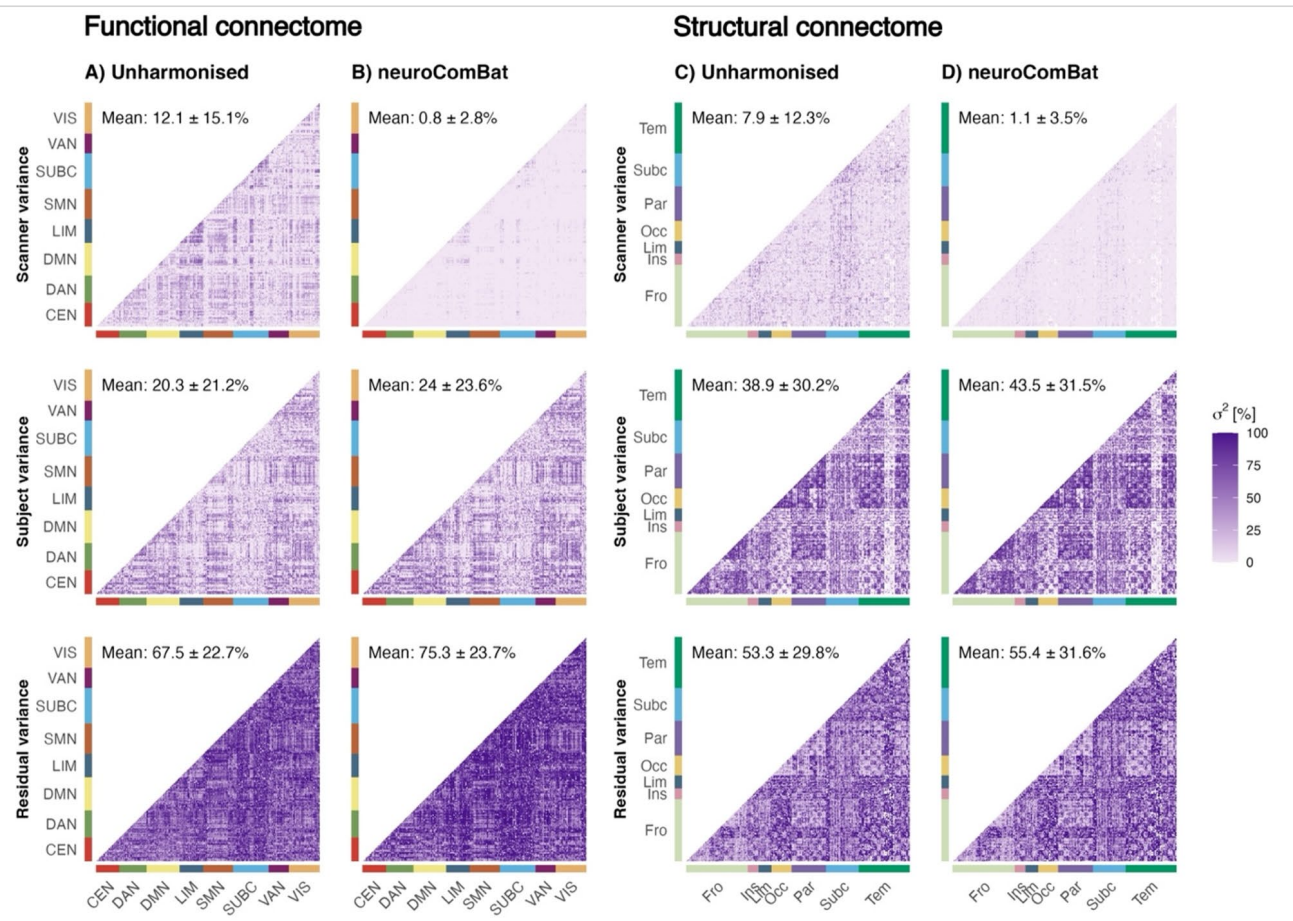
Results of the G Theory analysis for the functional (**A**,**B**) and structural (**C**,**D**) connectome. Each row depicts one variance component: scanner, subject and residual.

In the unharmonised functional connectome, 20.3 ± 21.2% of the variability was attributed to true inter-subject differences (Fig. 5A). The scanner was, with 12.1 ± 15.1% of explained variance, a meaningful source of systematic error (Fig. 5A). However, most variance remained unexplained, with 67.5 ± 22.7% attributed to the model’s residuals (Fig. 5A).

When applying *neuroComBat*, the scanner variance was reduced to 0.8 ± 2.8% and the variance of true inter-subject differences increased to 24 ± 23.6% (Fig. 5B). When comparing the large-scale brain networks across the harmonised and unharmonised functional connectome, the LIM and SUBC showed lower subject variance and higher residual variance (Fig. 5A,B). Additionally, the LIM showed higher scanner variance (Fig. 5A,B).

In the unharmonised structural connectome, 38.9 ± 30.2% of the variability was attributed to true inter-subject differences, 7.9 ± 12.3% to the variability of the scanner, yet most variance remained unexplained, with 53.3 ± 29.8% attributed to the model’s residuals (Fig. 5C). After *neuroComBat* harmonisation, the scanner variance was reduced to 1.1 ± 3.5% and the variance of true inter-subject differences increased to 43.5 ± 31.5% (Fig. 5D). When comparing anatomical regions across the harmonised and unharmonised structural connectome, the limbic and subcortical region showed higher scanner variance (Fig. 5C,D).

## Discussion

The study quantified cross-vendor reliability of FC and SC in healthy subjects at the edge-level between Siemens Prisma 3T and Philips Achieva 3.0T systems using a travelling cohort design and evaluated the impact of post-hoc *neuroComBat* harmonisation. In both FC and SC, the scanner was a meaningful source of systematic error. Reliability was better for (a) SC compared to FC, (b) group average data compared to individual subject data and (c) full connectome organisation compared to individual edges. *NeuroComBat* successfully mitigated scanner-dependent variance at the group but not the subject level.

### Scanner was a meaningful source of systematic error

The Generalisability Study of the edge-level FC and SC revealed that the scanner effects accounted for 11.8% and 7.9% of the variance in FC and SC, respectively. This represents a greater source of variability than that observed in a previous FC multisite study using Siemens and GE MRI systems across eight sites (2.5%)^6^. The scanner effect in our study includes effects solely attributable to the vendor, such as reconstruction algorithms, acceleration techniques, and field inhomogeneities but also includes sequence protocol differences and hardware variations. Hence, the imperfect sequence harmonisation and use of different head coils may have increased the scanner-dependent effect^25^. These results promote the urgency to carefully harmonise sequences across scanners and additionally use appropriate post-hoc harmonisation techniques in multisite studies.

Spatial differences in reliability emerge both in FC and SC: Subcortical and limbic regions were least reliable, with more variability attributed to scanner effects in SC and FC, and for FC also to the residuals. When inspecting the raw images (see Suppl. Fig. S1), greater b1 inhomogeneities and lower signal-to-noise ratio were visible in the subcortical and limbic regions of the Siemens images, which might have inflated scanner variability in said regions.

### Unravelling the unexplained variability

While subject effects explained 20.5% and 28.9% of the variance in FC and SC, the largest proportion of variance was attributable to the residual effect (FC 67.7%, SC 53.3%, unharmonised). The residual variance in our study reflects the variability across all scans that is not accounted by the main effects of subject and scanner. For example, this includes thermal fluctuations and random noise, which vary over time and may occur even within the same scanner. Similar proportions of residual variance (62.7%) have been reported in FC^6^. The authors are not aware of any comparable G Theory studies of SC.

#### Functional dynamics may drive unexplained variability in FC

The different reliability profiles of SC and FC observed in this study can be further interpreted within contemporary frameworks of systems neuroscience, where SC represents a stable anatomical scaffold, whereas FC captures dynamically evolving functional states shaped by spontaneous slow oscillations (SSO)^26^.

Consistent with previous studies on FC reliability, the subject-level reliability was low^27^. Paired with the high residual variance in FC, this aligns with the dynamic nature of intrinsic brain activity. Low edge-level reliability may partly reflect genuine temporal variability rather than measurement error alone as FC is not a fixed trait but a fluctuating reflection of ongoing spontaneous processes^28,29^. Measures of dynamic functional connectivity, as opposite to the static measure used in this study, may yield higher reliability by reducing the residual variance.

The higher reliability of within-network compared to between network FC further aligns with a hierarchical SSO model, where networks, such as DMN, CEN, and VAN, may exhibit more stable slow oscillatory coupling at the investigated frequency band^26,30^.

#### Methodological rather than biological instability in SC

The macroscale structural organisation of healthy adults’ brains is known to remain stable, especially in healthy participants over a short time period^31,32^. Against this background, the substantial unexplained variance observed in SC is unlikely to reflect genuine biological change in white matter architecture. Instead, it more plausibly points to methodical-related sources of variability.

This was surprising, as we employed a state-of-the-art structural connectivity pipeline, using a multi-shell multi-tissue constrained spherical deconvolution (MSMT-CSD) model with anatomically constrained probabilistic tractography. This approach was reported to have high accuracy and reliability compared to deterministic and DTI-based approaches^33^. By applying *SIFT2* to the tractogram, the connectome was weighted based on the underlying FOD amplitudes, ensuring correct proportionality among different connection pathways within a single subject, but not necessarily between subjects. We encountered this problem by setting an identical number of streamlines for each tractogram. However, estimating a group response function for FOD computation might yield better comparability between subjects and may reduce residual variance^20^.

Importantly, despite the high residual variance at the edge-level, SC demonstrated excellent pattern similarity. This suggests that the low edge-level reliability in SC is an additive or scaling effect (magnitude difference) rather than a fundamental difference in the spatial organisation of streamline reconstruction. This aligns with the theory, that the structural connectome provides a stable architecture. Lower reliability in specific limbic and subcortical edges is unlikely to reflect biological instability and may instead arise from tractography challenges in anatomically complex regions^34^, or scanner effects.

### Acceptable group-level reliability after harmonisation

The choice of analysis level markedly influences its reliability. It is a common finding in neuroimaging that group-level and full connectome analysis are more reliable than subject-level and edge-level analysis^27,35^.

The use of *neuroComBat* proved highly effective in reducing scanner-dependent variance to 0.8% and 1.1% in FC and SC, respectively. This is mirrored in excellent pattern similarity and minor overall bias after harmonisation, supporting acceptable group-level reliability. Despite the effective reduction of scanner-dependent variance achieved with *neuroComBat*, we nevertheless recommend including scanner as a covariate in group-level statistical models to account for any remaining scanner-related effects.

As neuroComBat is designed to align distributions of features across batches while preserving the relative rank order of subjects within a batch, its influence on subject-level reliability and similarity is inherently constrained. This is particularly true in situations where subject-level reliability is largely attributable to unexplained variance rather than systematic scanner-related bias. Our results are consistent with this limitation of post-hoc harmonisation, showing no improvement in subject-level reliability after applying *neuroComBat*.

### Limitations

The study must be interpreted with several limitations in mind. First, the small sample size (*n* = 10) constrains the stability of the estimated variance components and inflates uncertainty in the ICC estimates, including the occurrence of negative values – particularly in the presence of substantial within-subject variability as commonly observed in resting-state FC^35^. This limitation is especially relevant for FC, where the high residual variance and low ICCs may partly reflect insufficient sampling of each participant’s intrinsic activity repertoire rather than measurement error alone. Resting-state FC is known to stabilise with longer scan duration, aggregation across sessions and higher sampling rates^6,36–38^. Consequently, the true reliability across the full functional connectome may be higher than reported.

Second, the study’s focus on reliability between a Siemens Prisma 3T and a Philips Achieva 3.0T scanner limits the generalisability of the findings to other scanners or imaging sequences.

Third, the use of each scanner’s T1w image in the fMRI and dMRI pipeline may have introduced a systematic source of variance due to potential differences in registration. Future studies should use a common anatomical space between scanners to tackle this issue.

Finally, study design does not allow to differentiate subject-dependent retest effects (‘session effects’) from scanner-dependent effects and the residual variance includes both random measurement error and subject-scanner interactions. Addressing these issues would require a two-facet G Theory approach with four measurements per subject, two on each scanner. While this limitation is relevant for resting-state FC, given the expected fluctuations in FC over a one-week interval, it can be disregarded for SC, as the macroscale anatomical organisation of the brain remains stable within a week in healthy individuals. Nonetheless, future studies should aim for repeated runs within the same scanner to disentangle these effects.

## Conclusion

The differential reliability profiles of SC and FC can be explained by their unique organisation; SC reflects a rather stable anatomical backbone, and FC captures dynamic intrinsic processes. The results accentuate critical considerations for multi-centre studies and centres facing a scanner switch, as SC and FC are vulnerable to scanner-dependent effects. Consequently, researchers should (a) acquire bridging data on both scanners, when possible, (b) prioritise consistent hardware and software configurations (Tool example for sequence harmonisation: https://pulseq.github.io^39^, (c) interpret subject-level and edge-level connectome metrics, particularly containing subcortical and limbic regions, with caution. Post-hoc harmonisation techniques can be applied to SC and FC to restore comparability for group-level analyses – however, they are not designed to recover subject-level stability.

## Supplementary Information

Below is the link to the electronic supplementary material.

Supplementary Material 1

## Data Availability

Data and code used in this study are publicly available at (10.5281/zenodo.18769049).
