# Supplementary material for: Cross-vendor reliability of functional and structural brain connectivity in a travelling cohort
Source: Sci Rep. 2026 Apr 10;16:12071. doi: 10.1038/s41598-026-47705-1 (PMC13070028; doi:10.1038/s41598-026-47705-1)
Supplement: Supplementary file 1 — Supplementary Material 1 [file 41598_2026_47705_MOESM1_ESM.pdf]

Guidelines for Reporting Reliability and Agreement Studies (GRRAS)

Table 1: *GRRAS-Checklist*

No.	Item	Description	Stated where?
**TITLE AND ABSTRACT**			
1	Title/Abstract	Identify in title or abstract that interrater/intrarater reliability or agreement was investigated	Title
**INTRODUCTION**			
2	Measurement device	Name and describe the diagnostic or measurement device of interest explicitly	Introduction
3	Subject population	Specify the subject population of interest	Introduction
4	Rater population	Specify the rater population of interest (if applicable)	Introduciton
5	Rational	Describe what is already known about reliability and agreement and provide a rationale for the study (if applicable)	Introduction
**METHODS**			
6	Sample size	Explain how the sample size was chosen. State the determined number of raters, subjects/objects, and replicate observations	Methods/2.1
7	Sampling	Describe the sampling method	Methods/2.1
8	Measurement process	Describe the measurement/rating process (e.g. time interval between repeated measurements, availability of clinical information, blinding)	Methods/2.2–2.4
9	Independent measures	State whether measurements/ratings were conducted independently	Methods/2.2
10	Statistics	Describe the statistical analysis	Methods/2.5
**RESULTS**			
11	Number of datapoints included	State the actual number of raters and subjects/objects which were included and the number of replicate observations which were conducted	Results/3.1
12	Sample characteristics	Describe the sample characteristics of raters and subjects (e.g. training. experience)	Results/3.1
13	Estimates	Report estimates of reliability and agreement including measures of statistical uncertainty	Results/3.2–3.5
**DISCUSSION**			
14	Practical relevance	Discuss the practical relevance of results	Discussion
**AUXILIARY MATERIAL**			
15	Detailed results	Provide detailed results if possible (c.g. online)	Supplements

Supplementary data

Table 2: *Detailed description of used measures.*

Metric	Equation
Intraclass correlation coefficient (ICC)	$$\frac{MS_S - MS_E}{MS_S + (k-1)MS_E}$$ MS_S = mean square for subjects; MS_E = mean square for error; k = number of scanners
Within-subject coefficient of variation (wsCV)	$$\frac{SD_{ws}}{\bar{X}_{ws}} \times 100$$ SD_{ws} = standard deviation of one subject across scanners; $\bar{X}_{ws}$ = mean of one subject across scanners
Level of agreement (LoA)	$$MD \pm 1.96 \times SD$$ MD = mean difference; SD = standard deviation of the difference

Table 3: *Population characteristics per subject.*

Subject	Age (years)	BMI (kg/m^2)	Sex (M/F)	Days between measurements	MRI experience	First scan
sub-01	23	22.50	F	4	yes, no cMRI	SIEM
sub-02	33	23.24	M	6	yes	SIEM
sub-03	27	20.76	F	7	yes, no cMRI	PHIL
sub-04	24	28.40	M	7	yes, no cMRI	PHIL
sub-05	30	25.47	M	7	none	PHIL
sub-06	28	28.73	F	7	none	PHIL
sub-07	28	21.88	F	7	none	PHIL
sub-08	29	24.28	M	4	none	PHIL
sub-09	24	17.65	F	0	yes	PHIL
sub-10	26	21.01	M	1	yes	PHIL

Note. BMI = Body mass index. cMRI = Cranial magnetic resonance imaging. F = Female. M = Male. SIEM = Siemens Prisma 3T. PHIL = Philips Achieva 3.0T.

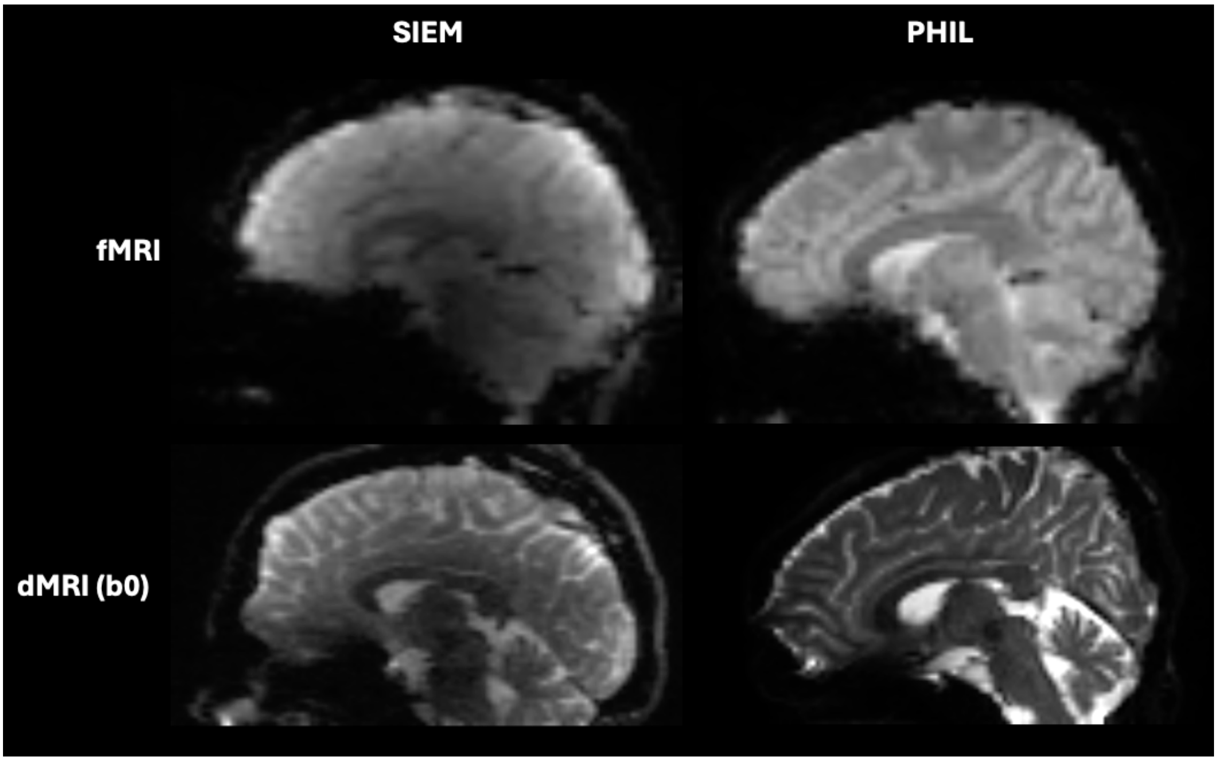

Figure S1: *Comparison of raw functional MRI (fMRI) and diffusion-weighted MRI (dMRI) images of a representative participant (sub-10) between the Siemens Prisma 3T (SIEM) and the Philips Achieva 3T (PHIL). The SIEM images show higher b1 homogeneities and lower signal-to-noise ratio in the subcortical and limbic regions, and overall lower grey matter-white matter contrast.*
